# Expansion of the calcium hypothesis of brain aging and Alzheimer's disease: minding the store

**DOI:** 10.1111/j.1474-9726.2007.00295.x

**Published:** 2007-06

**Authors:** Olivier Thibault, John C Gant, Philip W Landfield

**Affiliations:** Department of Molecular and Biomedical Pharmacology, University of Kentucky, University of Kentucky Medical Center Lexington, KY 40536, USA

**Keywords:** CICR, hippocampus, imaging, IP_3_, L-type Ca^2+^ channels, ryanodine receptor

## Abstract

Evidence accumulated over more than two decades has implicated Ca^2+^ dysregulation in brain aging and Alzheimer's disease (AD), giving rise to the Ca^2+^ hypothesis of brain aging and dementia. Electrophysiological, imaging, and behavioral studies in hippocampal or cortical neurons of rodents and rabbits have revealed aging-related increases in the slow afterhyperpolarization, Ca^2+^ spikes and currents, Ca^2+^ transients, and L-type voltage-gated Ca^2+^ channel (L-VGCC) activity. Several of these changes have been associated with age-related deficits in learning or memory. Consequently, one version of the Ca^2+^ hypothesis has been that increased L-VGCC activity drives many of the other Ca^2+^-related biomarkers of hippocampal aging. In addition, other studies have reported aging- or AD model-related alterations in Ca^2+^ release from ryanodine receptors (RyR) on intracellular stores. The Ca^2+^-sensitive RyR channels amplify plasmalemmal Ca^2+^ influx by the mechanism of Ca^2+^-induced Ca^2+^ release (CICR). Considerable evidence indicates that a preferred functional link is present between L-VGCCs and RyRs which operate in series in heart and some brain cells. Here, we review studies implicating RyRs in altered Ca^2+^ regulation in cell toxicity, aging, and AD. A recent study from our laboratory showed that increased CICR plays a necessary role in the emergence of Ca^2+^-related biomarkers of aging. Consequently, we propose an expanded L-VGCC/Ca^2+^ hypothesis, in which aging/pathological changes occur in both L-type Ca^2+^ channels and RyRs, and interact to abnormally amplify Ca^2+^ transients. In turn, the increased transients result in dysregulation of multiple Ca^2+^-dependent processes and, through somewhat different pathways, in accelerated functional decline during aging and AD.

## Introduction

It has been over 20 years since it was initially proposed that altered Ca^2+^ regulation might play a role in brain aging and Alzheimer's disease (AD) (Landfield, 1983, 1987; Khachaturian, 1984, 1989; Gibson & Peterson, 1987; Disterhoft *et al*., 1994). In brain neurons from aging rodents and rabbits, as compared to neurons from younger animals, Ca^2+^ influx associated with action potentials induces a larger Ca^2+^-dependent afterhyperpolarization (AHP) (Landfield & Pitler, 1984; Kerr *et al*., 1989; Moyer *et al*., 1992; Potier *et al*., 1992; Disterhoft *et al*., 1996, 2004; Stutzmann *et al*., 2006) and impairs short-term synaptic plasticity (Landfield *et al*., 1986; Thibault *et al*., 2001). Furthermore, these findings have been reinforced by studies showing that pharmacologically isolated Ca^2+^ action potentials (Pitler & Landfield, 1990; Disterhoft *et al*., 1996), whole-cell Ca^2+^ currents (Campbell *et al*., 1996), and Ca^2+^ transients during repetitive spike trains also are larger in hippocampal neurons from aged animals (Thibault *et al*., 2001; Hemond & Jaffe, 2005). Conversely, Ca^2+^ influx via ligand-gated N-methyl-D-aspartate (NMDA) receptor channels appears reduced in aged animals (Barnes *et al*., 1997; Magnusson, 1998; Shankar *et al*., 1998).

Our studies on this general Ca^2+^ dysregulation hypothesis have focused primarily on apparent excess Ca^2+^ influx via voltage-gated Ca^2+^ channels (VGCC) (Landfield, 1996; Thibault *et al*., 1998). Studies of the L-type VGCC (L-VGCC) antagonist suggested that the aging-related increase in Ca^2+^-mediated responses might depend on greater activity through L-VGCC (Moyer *et al*., 1992; Campbell *et al*., 1996). Increased L-VGCC activity with aging was confirmed directly by single channel recording in partially dissociated hippocampal slices (Thibault & Landfield, 1996). Moreover, changes in L-VGCCs appear to be functionally relevant, as L-VGCC antagonists improve learning and memory in aged animals (Deyo *et al*., 1989; Disterhoft *et al*., 2004) and some AD patients (Forette *et al*., 2002). Furthermore, the increase in L-VGCC density is positively correlated with cognitive impairment in aged animals (Thibault & Landfield, 1996).

In addition to the accumulating evidence of increased Ca^2+^ influx through L-VGCCs, there is also recent evidence that altered function of intracellular organelles might play a critical role in Ca^2+^ regulation during aging or AD (Toescu & Verkhratsky, 2003). In particular, changes in intracellular Ca^2+^ release from the endoplasmic reticulum (ER) appear likely to contribute to brain Ca^2+^ dyshomeostasis, and have been associated with changes in [Ca^2+^]_i_. Therefore, in this review, we summarize several lines of evidence implicating altered release from intracellular stores in aging and AD, and attempt to integrate this evidence with the role of Ca^2+^ influx in aging-related Ca^2+^ dysregulation.

## Interactions between L-VGCCs and Ca^2+^-induced Ca^2+^ release from the endoplasmic/sarcoplasmic reticulum

Several comprehensive reviews have recently considered mechanisms associated with Ca^2+^ sequestration and release by the ER in both peripheral cells (Bootman *et al*., 2001; Berridge, 2002; Carafoli, 2002; Fill & Copello, 2002) and in neurons (Paschen & Mengesdorf, 2005; Verkhratsky, 2005). Accordingly, only the points most relevant to ER function in brain aging are briefly recapitulated here. Two distinct intracellular Ca^2+^ release channels are present in several types of muscle and brain cells, the inositol 1,4,5-trisphospate receptor (IP_3_R) and the ryanodine receptor (RyR), each having multiple isoforms in different tissues. These receptor channels function to amplify or trigger Ca^2+^ rises initiated by either plasmalemmal Ca^2+^ influx or ligand binding, thereby inducing Ca^2+^ signaling cascades. Amplification is achieved through either the actions of Ca^2+^-induced Ca^2+^ release (CICR), provided by RyR, or actions of IP_3_-induced Ca^2+^ release (IICR) through IP_3_Rs.

Originally described in skeletal and cardiac muscle cells, RyRs in the membrane of the sarcoplasmic reticulum are an integral and essential Ca^2+^ source for excitation-contraction coupling (Endo, 1977; Fill *et al*., 1989; Takeshima *et al*., 1989; Meissner, 1994). Furthermore, an apparent direct physical interaction, which favors alignment between L-VGCCs and RyRs, enables L-VGCCs to function as a preferred source of extracellularly derived Ca^2+^ in triggering CICR from RyRs and amplifying Ca^2+^ transients (Lu *et al*., 1994; Cheng *et al*., 1996; Wang *et al*., 2001). In the brain, similar Ca^2+^ amplification functions of RyRs have been identified, again mediated in part by a close juxtaposition to L-VGCCs (Chavis *et al*., 1996; Empson & Galione, 1997; Borde *et al*., 2000; Fagni *et al*., 2000; Sukhareva *et al*., 2002).

The other major source of intracellular Ca^2+^ occurs in response to stimulation of IP_3_Rs by IP_3_ generated from activation of a number of metabotropic G-protein-coupled receptors. In some cases IP_3_Rs can also trigger Ca^2+^-sensitive K^+^ channels and hyperpolarize neurons (Sawada *et al*., 1987; Fink *et al*., 1988; Furuichi *et al*., 1989; Zhang *et al*., 1990; Berridge, 1993; Khodakhah & Ogden, 1995; Irving & Collingridge, 1998; Taylor *et al*., 1999; Johenning *et al*., 2002; Rossi & Taylor, 2004). Moreover, IP_3_Rs are also sensitive to Ca^2+^ concentrations (Bezprozvanny *et al*., 1991; Missiaen *et al*., 1992; Tsukioka *et al*., 1994; Hagar *et al*., 1998) and, depending on the cell type studied, it appears that IP_3_R may also be favorably aligned with L-VGCCs or metabotropic glutamate receptors (mGluR), through interactions with the scaffold protein Homer 1a (Tu *et al*., 1998; Fagni *et al*., 2000; Yamamoto *et al*., 2005).

Release of Ca^2+^ from these two intracellular channels is regulated in part by the Ca^2+^ concentration gradient present between luminal ER Ca^2+^ and cytoplasmic Ca^2+^ (Alonso *et al*., 1999; Kiryushko *et al*., 2002; Solovyova *et al*., 2002) and is, thus, also dependent on the Ca^2+^-refilling function of sarcoplasmic/endoplasmic reticulum Ca^2+^-ATPases (SERCA). Sarcoplasmic/endoplasmic reticulum Ca^2+^-ATPases maintain the relatively high levels of Ca^2+^ in the ER (hundreds of µm) that serve CICR, and IICR, and, in the process, contribute to the control and reduction of cytosolic Ca^2+^ (Thastrup *et al*., 1990; MacLennan *et al*., 1997; Mogami *et al*., 1998; Meldolesi, 2001; Berridge, 2002; Verkhratsky, 2004).

## Dysregulated Ca^2+^ and ER function in models of ischemia and toxicity

Although cell culture models of Ca^2+^-dependent cell death are generally not viewed as clear models of brain aging, or even AD, they are often employed in studies of ischemic events. These events increase in frequency with advancing age, and it is also possible that neuronal vulnerability from such events increases with aging. Therefore, examining the role of Ca^2+^ release from intracellular stores in cell death models may help elucidate implications of aging-related alterations in intracellular release. In particular, delayed toxicity after exposure to high glutamate (GLU) in cell culture (excitotoxicity) is a common model used to mimic a wide range of neurological insults, including anoxia/ischemia, head and spinal cord trauma, and even chronic neurodegenerative diseases such as AD. Dysregulated Ca^2+^ homeostasis and altered Ca^2+^ influx through NMDA receptors were identified as primary contributors to neuronal cell death early in the study of excitotoxicity (Rothman & Olney, 1986; Choi *et al*., 1987; Wahl *et al*., 1989; Regan & Choi, 1991; Randall & Thayer, 1992; Dubinsky, 1993; Lu *et al*., 1994; Marks *et al*., 1996; Tymianski & Tator, 1996; Toescu, 1998; Lee *et al*., 1999; Limbrick *et al*., 2001; Lipton, 2004). In excitotoxicity models, Ca^2+^ dysregulation is frequently manifested as an irreversible Ca^2+^ rise or slowed Ca^2+^ clearance, and is ultimately associated with neuronal death.

Several investigations of excitotoxicity have focused on a potential role of the ER in sustained Ca^2+^ elevations. These studies have found that blocking CICR with high concentrations of ryanodine, which lock RyRs in a low conductance state (Bezprozvanny *et al*., 1991; Coronado *et al*., 1994; Humerickhouse *et al*., 1994), or irreversibly inhibiting SERCA function and passively emptying ER stores with thapsigargin prior to GLU exposure, reduces sustained Ca^2+^ plateaus, as well as other indices associated with neuronal cell death (e.g. lactate dehydrogenase (LDH) release) (Frandsen & Schousboe, 1991; Segal & Manor, 1992; Leski *et al*., 1999; Clodfelter *et al*., 2002). Similar results have been noted in models of stroke and ischemia, particularly in astrocyte preparations (Duffy & MacVicar, 1996; Kuwabara *et al*., 1996; Verkhratsky *et al*., 1998; Aley *et al*., 2006). Somewhat paradoxically, while short-term ER Ca^2+^ depletion prior to an insult appears protective against necrotic (excitotoxic) cell death, long-term depletion of ER Ca^2+^ induces apoptosis, as indicated by elevations of apoptotic markers, stress responses and disturbance in protein synthesis, and/or massive cell death (Doutheil *et al*., 1999; Mengesdorf *et al*., 2001; Verkhratsky & Petersen, 2002; Paschen, 2003; Verkhratsky & Toescu, 2003; Lindholm *et al*., 2006).

**Fig. 1:**
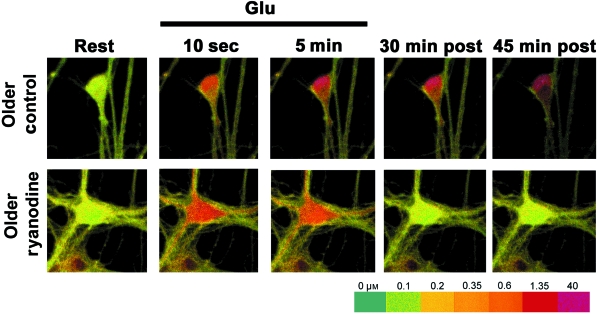
Ryanodine protection of older cultured hippocampal neurons from excitotoxicity. Following a glutamate insult, older cultured neurons exhibit a sustained [Ca^2+^]_i_ elevation leading to cell death. Confocal indo-1 Ca^2+^ imaging shows ryanodine facilitated the recovery (decline) of the Ca^2+^ plateau and protected older neurons following glutamate insult (modified from Clodfelter *et al.* copyright 2002 with permission from Elsevier).

Thus, excessive release of Ca^2+^ from the ER may play an important role in excitotoxicity. Moreover, evidence suggests that such excessive release may be dependent on the relative maturity of the cells. It is well established that embryonic cortical/hippocampal neurons become increasingly vulnerable to GLU toxicity after a few weeks in culture (Choi, 1992; Toescu & Verkhratsky, 2000), an age in culture that coincides with the emergence of sustained Ca^2+^ plateaus following GLU insult (Attucci *et al*., 2002). Interestingly, ryanodine is particularly effective in reversing the Ca^2+^ plateau and in providing neuroprotection in older cultures (Fig. 1) (Clodfelter *et al*., 2002). Moreover, recent evidence suggests that the lethal Ca^2+^ plateau may be maintained by sustained Ca^2+^ influx via depolarized NMDA receptors (Norris *et al*., 2006). Together, these data indicate that the plateau may be sustained by CICR. Although age in culture is clearly not equivalent to brain aging, it is associated with increasing vulnerability and Ca^2+^ influx, which may model some aspects of normal aging (Porter *et al*., 1997). Conceivably therefore, if Ca^2+^ release from ER is altered with aging, this alteration may develop in parallel with altered Ca^2+^ influx (Clodfelter *et al*., 2002).

## Ca^2+^ release from ER in models of AD

With the increasing development of transgenic (Tg) mouse models of AD, numerous studies testing the view that altered Ca^2+^ homeostasis might play a role in AD have recently emerged. Initial studies in fibroblasts from AD patients (Gibson *et al*., 1996) or in cells bearing the human presenilin 1 (PS1) AD mutation (Begley *et al*., 1999; Guo *et al*., 1999; Leissring *et al*., 1999; LaFerla, 2002; Stutzmann, 2005) found evidence of abnormal Ca^2+^ release through IP_3_R pathways (Leissring *et al*., 1999). Interestingly, abnormal IP_3_-mediated Ca^2+^ elevations in fibroblasts have also been seen in asymptomatic members of AD families (Etcheberrigaray *et al*., 1998).

Several studies also have implicated RyRs as being responsible for enhanced intracellular release in PS1 mutated animals (Chan *et al*., 2000; Mattson *et al*., 2000; Schneider *et al*., 2001; Popescu & Ankarcrona, 2004; Stutzmann *et al*., 2006). Smith and colleagues (2005) examined cultured cortical neurons from mice bearing a transgene containing three AD-related mutations (3×Tg mice), which develop both plaques and tangles, and observed an increase in RyR expression along with greater Ca^2+^ efflux from the stores in response to caffeine (an agonist at the RyR) (Smith *et al*., 2005). A recent study combining electrophysiological and Ca^2+^ imaging methods in cortical slice neurons from Tg mice bearing the PS1 mutation alone, or the 3×Tg transgene, or nontransgenic control animals, assessed the effects of aging vs. those of the PS1 mutation on ER release (Stutzmann *et al*., 2006). This study found that the PS1 mutation is a critical calciopathic mutation and that increased RyR expression is likely a major factor in the AD mutation-mediated enhancement of ER release. Although photolysis of IP_3_ was shown to evoke larger Ca^2+^ transients and Ca^2+^-dependent hyperpolarizations in Tg mice, the increase in IP_3_ effects was mediated by CICR from RyRs, triggered in response to IICR. However, some puzzling results also were seen. The enhanced IP_3_-mediated Ca^2+^ release and resulting hyperpolarization was larger in Tgs than in non-Tgs at all ages and did not change with aging in any Tg or non-Tg model. Conversely, the AHP induced by trains of spikes and VGCC activation increased with aging in all three model strains but did not differ between Tg and non-Tg mice (Stutzmann *et al*., 2006).

While little is known regarding underlying mechanisms, it appears that altered CICR, perhaps in combination with IICR, confer some of the phenotypes of disrupted Ca^2+^ homeostasis in neurons from 3×Tg mice. Still, other sources and mechanisms likely also contribute. The PS1 mutation (which, alone, does not induce amyloid plaques), in combination with amyloid precursor protein (APP) mutations, increases Aβ production (Mullan & Crawford, 1993; Price & Sisodia, 1994; Tanzi *et al*., 1996; Holcomb *et al*., 1998; Selkoe, 1998). Some studies have found that Aβ production can exacerbate Ca^2+^ responses to NMDA or GLU exposure (Mattson, 1997). Furthermore, Aβ toxicity has been attributed, in part, to effects on VGCCs (Davidson *et al*., 1994; Weiss *et al*., 1994; Ueda *et al*., 1997; Ramsden *et al*., 2002; Bobich *et al*., 2004; Webster *et al*., 2006), which could trigger CICR from IP_3_Rs or RyRs (Koizumi *et al*., 1998; Ferreiro *et al*., 2004). However, APP proteolysis (γ-secretase activity) alone does not appear sufficient, because the PS1 mutation (rather than other more amyloidogenic mutations) must be present for the Ca^2+^ dysregulation to occur (Stutzmann *et al*., 2006). A possible alternative mechanism suggests that presenilins form Ca^2+^ leak channels in ER membranes of mouse fibroblasts, independently of γ-secretase activity. Mutations in presenilin interfere with this leak function, and result in greater Ca^2+^ filling and release from ER (Tu *et al*., 2006). Furthermore, a gene microarray study conducted in autopsied hippocampal tissue from human AD patients (Blalock *et al*., 2004) found that multiple genes encoding proteins involved in ER receptor function, or in protein folding and chaperoning, which are also mediated in part by the ER, were down-regulated in incipient AD. These widespread changes may reflect ER membrane/receptor instability in sporadic AD as well.

In addition, it should be noted that effects of PS1 mutations on Ca^2+^ dysregulation have been observed to occur via other processes, including capacitative Ca^2+^ entry (Yoo *et al*., 2000; Smith *et al*., 2002; Herms *et al*., 2003; Zatti *et al*., 2006), changes in mitochondrial potential (Begley *et al*., 1999; Ankarcrona & Hultenby, 2002; Chan *et al*., 2002; Behbahani *et al*., 2006), and L-VGCCs (Cook *et al*., 2005). Clearly therefore additional work will be needed to resolve the relative contributions of the different sources to the Ca^2+^dysregulation seen in various models of neurodegenerative diseases.

## Neuronal ER release in normal aging

Electrophysiological markers of brain aging have been extensively characterized in the hippocampal formation (Landfield & Pitler, 1984; Moyer *et al*., 1992; Barnes, 1994; Thibault *et al*., 1998; Norris *et al*., 1998; Disterhoft *et al*., 2004; Burke & Barnes, 2006), a region well-established to be important for memory processes and highly vulnerable to deleterious/degenerative changes with aging. Many of the consistent biomarkers of aging, such as the slow AHP (sAHP), are Ca^2+^-dependent or Ca^2+^-mediated. However, it is important to assess the degree to which the ER contributes to the established biomarkers of aging. Both CICR and IICR pools exist within the ER of hippocampal CA1 and CA3 pyramidal neurons. The amount of Ca^2+^ released via CICR and IICR depends on binding of intracellular ligands including Ca^2+^, cyclic ADP ribose (cADPR), nicotinic acid adenine dinucleotide phosphate (NAADP) or IP_3_ (Verkhratsky, 2005), and also depends on the Ca^2+^ sequestering capacity of the ER, which determines ER Ca^2+^ content ([Ca^2+^]_L_) (Verma *et al*., 1992; Murayama & Ogawa, 1996; Dawson, 1997; Garaschuk *et al*., 1997). Solovyova and colleagues using a dual indicator loading technique (low affinity indicator for imaging Ca^2+^ in the ER, and high affinity indicator for imaging Ca^2+^ in the cytosol) were able to show that the resting [Ca^2+^]_L_ in sensory neurons is in the range of 200–300 µm, and high concentrations of IP_3_ or caffeine result in approximately a 40% decrease in luminal Ca^2+^ (Solovyova *et al*., 2002). Depolarization induced [Ca^2+^]_L_ release was less effective, ranging from 5 to 30 µm. Other techniques for imaging Ca^2+^ within the ER include the use of aequorin or cameleons. However, there are limitations with these techniques, as the Ca^2+^ reporting proteins must be genetically engineered and selectively targeted to the ER (Miyawaki *et al*., 1997; Alonso *et al*., 1998; Solovyova & Verkhratsky, 2002). In addition, they require long incubation times for transfecting and loading and, thus, preclude their use in acute brain slices.

Consequently, there have been only a handful of studies in neurons examining the effects of aging on ER Ca^2+^ concentration and release, or on RyR expression. Studies focusing on measures of ER Ca^2+^ content have generally relied on the use of single wavelength indicators to measure changes in [Ca^2+^]_i_ transients activated by caffeine, and have found varying results, depending on the experimental approach or preparation. In an early study, no net change in ER Ca^2+^ release with aging was reported in synaptosomes from the whole brain (Martinez-Serrano *et al*., 1992). More recently, acute dissociation of several brain regions (cerebellar, basal forebrain, and hippocampal neurons) from aged animals found that CICR magnitude was reduced and that Ca^2+^ transients recovered more slowly (Verkhratsky *et al*., 1994; Kirischuk & Verkhratsky, 1996; Murchison & Griffith, 1999; Xiong *et al*., 2002; Alshuaib *et al*., 2006). In studies focusing on RyR expression, no clear pattern or consistent changes have been seen in neurons of normal aging rats and mice. Two studies reported no change in brain RyR expression during aging (Martini *et al*., 1994; Stutzmann *et al*., 2006), although a recent study of peripheral neurons found a transient elevation in protein levels (RyR3) in mid-aged rats (Vanterpool *et al*., 2006).

Another approach to the investigation of the possible role of the ER in brain aging is to examine the effects of aging on Ca^2+^-dependent processes that are modulated, in part, by intracellular Ca^2+^ release. In CA1 neurons, postsynaptic injection of IP_3_ or of RyR inhibitors prevents the induction of long-term potentiation and attenuates paired-pulse facilitation (Wang & Kelly, 1997). Similarly, bath application of thapsigargin or cyclopiazonic acid (blockers of SERCA) prevents the induction of long-term depression in both single neurons and in field potential measures (Reyes & Stanton, 1996). High concentrations of ryanodine also selectively reduce the sAHP and spike-frequency accommodation (Borde *et al*., 2000; Shah & Haylett, 2000). While examining the effect of aging on long-term depression induction (Norris *et al*., 1998), Foster and colleagues recently reported that cyclopiazonic acid, thapsigargin or ryanodine (agents that reduce CICR) all prevented long-term depression in aged neurons (Kumar & Foster, 2005). However, long-term potentiation, which tends to be decreased with aging (Burke & Barnes, 2006), was enhanced by high ryanodine concentrations in aged slices (Kumar & Foster, 2004). Ca^2+^-dependent processes mediated largely by IICR and mGluRs activation also have been shown to change with aging. Compared to younger animals, type 1 mGluR activation results in a reduced phosphoinositide turnover in aged rats, perhaps mediated by a reduction in phospholipase C activity (Nicolle *et al*., 1999). Similarly, protein kinase C (PKC) was also reported to show reduced activity in aging neurons (Araki *et al*., 1994; Pascale *et al*., 1998).

Thus, the evidence on the nature of altered CICR or IICR in neurons of normally aging mammals is somewhat inconsistent, perhaps reflecting the type of preparation, cell or brain region specificity, or the difficulty in imaging Ca^2+^ and its sources within the intact hippocampal slice (Brown & Jaffe, 1994). Recently therefore we sought to systematically test the contributions of CICR to aging changes in one of the brain regions studied most extensively in relation to aging (hippocampus). Specifically, we tested the key prediction that, if increased CICR plays a major role in normal brain aging, then blocking it with high concentration ryanodine should reduce the aging differences in multiple Ca^2+^ biomarkers of aging.

More broadly, in fact, several other important tenets of the overall Ca^2+^ hypothesis have, for some time, required adequate testing. These tenets and predictions include: (i) if a common mechanism of Ca^2+^ dysregulation underlies many aspects of brain aging, then multiple Ca^2+^-dependent biomarkers of aging in the hippocampus should emerge at approximately the same age in adulthood; and (ii) if Ca^2+^ dysregulation is a major factor in cognitive decline then Ca^2+^ biomarkers should precede or coincide with the earliest age of cognitive impairment, which in some studies of rats has been as early as 12-months old (approximately mid-life). To test these predictions and the involvement of CICR on the emergence of Ca^2+^-related biomarkers, we recently conducted an extensive age course study combining electrophysiological and Ca^2+^ imaging techniques in hippocampal slices from male rats. Animals at five age points were used to identify the age of onset for three Ca^2+^-mediated markers of aging, the sAHP, spike accommodation, and the synaptically activated Ca^2+^ transient. A subset of hippocampal slices received a high dose of ryanodine to block the contribution of CICR to the overall Ca^2+^ response. In this study, we also employed the least invasive procedures available (sharp intracellular electrodes instead of patch clamping electrodes, nondissociated slices) to minimize interactions of preparation trauma and age.

**Fig. 2:**
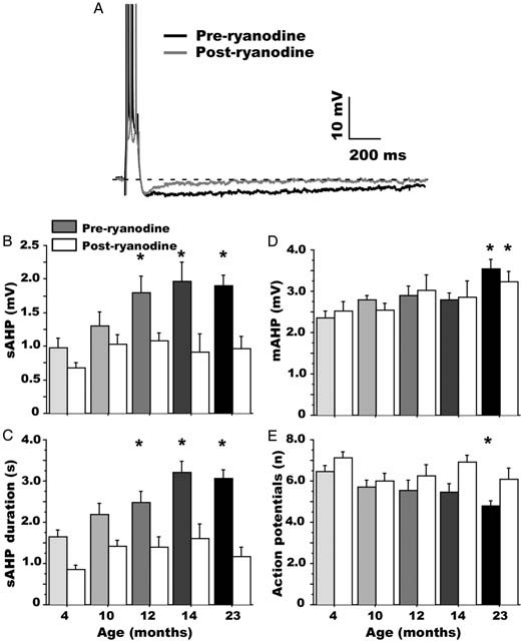
Ryanodine reduces the slow afterhyperpolarization (AHP) in an age-dependent manner. (A) Representative example of the blocking effect of 20 µm ryanodine on the AHP of a 23-month-old rat CA1 neuron. (B) Age dependence of slow AHP (sAHP) amplitude, before and following ryanodine application. (C) Age dependence of slow AHP duration, pre- and postryanodine. (D) Age dependence of medium AHP (mAHP) amplitude, pre- and postryanodine. (E) Age-dependence measures of spike-frequency accommodation, pre- and postryanodine. * indicates a significant difference from the 4-month-old group (*P <* 0.05). Note that aging changes in sAHP markers emerge at 12 months of age (preryanodine group), and ryanodine completely eliminates the aging effects (B and C), indicating a selective blockade of the aging-related increase in Ca^2+^-induced Ca^2+^ release (CICR). The initial mAHP is not modulated by CICR (A) and its age dependence was not altered by ryanodine (D). Action potential accommodation changes generally followed the sAHP pattern, but the aging effect at 12 months was not significant in this subset of cells (mean ± SEM) (from Gant *et al*. copyright 2006 with permission from the Society for Neuroscience).

**Fig. 3:**
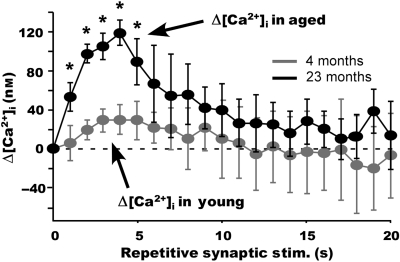
Ryanodine-sensitive component of the [Ca^2+^]_i_ rise during repetitive synaptic stimulation. Ca^2+^-induced Ca^2+^ release (CICR) contribution to the [Ca^2+^]_i_ rise was determined by subtracting [Ca^2+^]_i_ measures following ryanodine from those before ryanodine application (Δ[Ca^2+^]_i_), in neurons from 4- and 23-month-old animals during 20-s trains of 7 Hz suprathreshold synaptic stimulation. Values shown represent only the (CICR) component of the Ca^2+^ response that was blocked by ryanodine. Note that the ryanodine-sensitive component of [Ca^2+^]_i_ is significantly greater in aged rat neurons and contributes to the Ca^2+^ response primarily during the first 5 s of stimulation. * indicates a significant difference from the 4-month-old group (*P <* 0.05). (mean ± SEM).

Results were consistent with the above predictions. That is, ryanodine essentially eliminated aging differences in the three markers (e.g. the sAHP, Fig. 2), and the three biomarkers were first detectable simultaneously and at 12 months of age (Fig. 2), an age range early enough to account for cognitive decline. The ryanodine-sensitive component of the Ca^2+^ response (i.e. CICR) during a 20-s train of synaptic spikes appears to be minimal in young neurons compared to aged neurons and, notably, CICR contributed most to the [Ca^2+^]_i_ elevation during the first few seconds of the train (Fig. 3). This rapid ‘booster’ action of CICR on Ca^2+^ responses is consistent with its strong effect on the AHP (Fig. 2) (Gant *et al*., 2006).

**Fig. 4:**
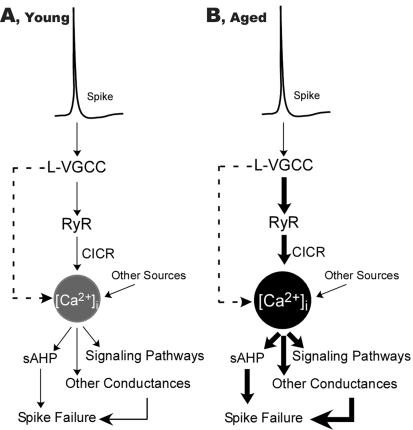
Schematic model of alterations in L-type voltage-gated Ca^2+^ channels (L-VGCC) and Ca^2+^-induced Ca^2+^ release (CICR) that drive other Ca^2+^-related hippocampal biomarkers of aging. With aging, increased L-VGCC activity and enhanced CICR operate in series, amplifying the impact of Ca^2+^ influx on multiple Ca^2+^-dependent functions. The thickness of arrows schematically represents the activity of Ca^2+^ flux or signaling pathways in aged rat neurons (B) relative to young (A). These pathways are increased at several stages despite equivalent spike amplitudes and durations. Dashed arrows indicate a possible direct parallel contribution of L-VGCCs to [Ca^2+^]_i_ (From Gant *et al*. copyright 2006 with permission from the Society for Neuroscience).

Thus, results of this large study provide considerable support for the proposition that in the hippocampus, an aging-related increase in CICR is necessary, from the onset, for the development of aging changes in several Ca^2+^-related processes. Moreover, the findings may help to resolve some of the contradictions in the literature by elucidating the conditions under which the contributions of CICR are most prominent. However, one apparent paradox is that similar kinds of evidence support a critical role for L-VGCCs in aging-related Ca^2+^ dysregulation (Thibault *et al*., 1998; Disterhoft *et al*., 2004). Nevertheless, these two lines of evidence are not necessarily contradictory, given that L-VGCCs and RyRs appear to operate in series in many cell types. In this view, then, Ca^2+^ influx via L-VGCCs may be the preferred source for triggering elevated CICR in aging. Together, the data suggest that aging changes in both types of channel may be part of the same pathway of dysregulation, in turn, suggesting the utility of expanding this version of the Ca^2+^ hypothesis to incorporate the results on Ca^2+^ release from intracellular stores (Fig. 4).

## Conclusions and a new model of Ca^2+^ dysregulation in hippocampal aging

The work summarized above points to the following basic conclusions:

1. Extensive evidence supporting the hypothesis that Ca^2+^ dysregulation contributes in part to brain aging and AD that has accumulated for more than 20 years, some of it implicating a larger Ca^2+^-dependent AHP and increased activity of L-type Ca^2+^ channels in the functional and cognitive decline seen with normal aging in mammals.
2. Elevated Ca^2+^ release from RyRs appears to contribute importantly to cell death and vulnerability in several models of toxicity, which may have relevance to aging-associated ischemic events or other degenerative conditions.
3. Some types of AD mutations (e.g. presenilins), but not all, appear to alter RyR expression. Under some conditions, (e.g. IP_3_ stimulation and consequent CICR), this can result in elevated intracellular Ca^2+^ release and greater hyperpolarization of cortical neurons from transgenic mice of all ages. Surprisingly, however, in the triple transgenic AD model, the aging-related increase in spike train-induced AHP did not differ from the aging change in the AHP seen in wild-type mice.
4. The observed contributions of altered CICR to Ca^2+^ dysregulation in neurons during normal aging have been somewhat inconsistent, apparently depending, in part, on cell type and preparation, regional localization and possibly species. However, our recent studies in hippocampal slices from rats of increasing age (five age points) indicate that elevated CICR, beginning at about 12 months of age, may be an important underlying factor in the emergence of multiple Ca^2+^-related biomarkers of brain aging in rats.
5. The apparent strong evidence linking both L-VGCCs and RyRs to dysregulated hippocampal Ca^2+^ homeostasis during aging, rather than being contradictory, may instead suggest an expanded model of the Ca^2+^ dysregulation pathway in brain aging and, perhaps in AD (as shown in Fig. 4). In this new model, L-VGCCs and RyRs operate in series and aging changes in both (or either) contribute to the aberrant amplification of Ca^2+^ transients.
